# Cable equation formalism for neuronal magnetic fields

**DOI:** 10.1186/1471-2202-15-S1-P215

**Published:** 2014-07-21

**Authors:** Alain Destexhe, Francesca Barbieri, Claude Bedard

**Affiliations:** 1 UNIC, CNRS, Gif sur Yvette, France

## 

Neurons generate magnetic fields which can be recorded with macroscopic techniques such as magneto-encephalography. The theory that accounts for the genesis of neuronal magnetic fields involves macroscopic dipole structures in homogeneous resistive extracellular media. Here, we study this problem at the microscopic level using a variant of cable theory which accounts for the genesis of magnetic fields, in extracellular media with arbitrarily complex electric properties.

The cable formalism, initially introduced by Rall [1], has been recently generalized to include the influence of the extracellular medium [2]. We use here this generalized cable formalism to calculate the magnetic field generated by neurons. We show that the magnetic induction generally depends on the impedance of the extracellular and intracellular media. Therefore, like the electric field, the electric properties of these media can influence the magnetic field, contrary to what is usually assumed.

Next, we use this formalism to calculate the "magnetic signature" of different neuronal morphologies, such as pyramidal cells and basket cells. We show that the strongest magnetic fields correspond to media which are non-resistive, such as diffusive media, although they exert low-pass filtering properties. Recent measurements (see companion poster by Bedard et al.) suggest that the extracellular medium is best described by a diffusive impedance. We therefore predict that this will also affect neuronal magnetic fields.

In conclusion, we show here that the generalized cable formalism is an important tool to calculate the extracellular electric and magnetic fields generated by neurons. The nature of the medium influences both types of fields, and should be measurable by appropriate measurements. Given the fact that the nature of the medium influences the magnetic field, inverse methods should consider this important parameter.
